# Association Between Physical Activity and Pancreatic Cancer Risk and Mortality: A Systematic Review and Meta-Analysis

**DOI:** 10.3390/cancers16213594

**Published:** 2024-10-24

**Authors:** Mylena D. Bos, Jelmer E. Oor, Lucas Goense, N. Helge Meyer, Maximilian Bockhorn, Frederik J. H. Hoogwater, Joost M. Klaase, Maarten W. Nijkamp

**Affiliations:** 1 Department of Surgery, Division of Hepato-Pancreato-Biliary Surgery and Liver Transplantation, University Medical Center Groningen, University of Groningen, 9713 GZ Groningen, The Netherlands; m.d.bos@umcg.nl (M.D.B.); f.j.h.hoogwater@umcg.nl (F.J.H.H.); j.m.klaase@umcg.nl (J.M.K.); 2 Department of Surgery, University Medical Center Utrecht, Utrecht University, 3584 CX Utrecht, The Netherlands; j.e.oor@umcutrecht.nl (J.E.O.); l.goense@umcutrecht.nl (L.G.); 3 Department of Human Medicine, University Hospital of General and Visceral Surgery, University of Oldenburg and Klinikum Oldenburg, 26133 Oldenburg, Germany; helge.meyer@uni-oldenburg.de (N.H.M.); maximilian.bockhorn@uni-oldenburg.de (M.B.)

**Keywords:** pancreatic cancer, physical activity, risk, mortality

## Abstract

**Background:** Physical activity has been associated with a lower risk of various types of cancer and reduced cancer-specific mortality. Less is known about its impact on pancreatic cancer. The aim of this systematic review and meta-analysis was to summarize evidence on the association between physical activity and pancreatic cancer risk and mortality. **Methods**: PubMed and Embase were searched until May 2024 for studies examining physical activity in relation to pancreatic cancer incidence and mortality. Summary risk estimates for highest vs. lowest physical activity levels were calculated using a random-effects model. The risk of publication bias was assessed with a funnel plot and Egger’s regression test. **Results**: A total of seven case–control and eighteen prospective cohort studies were included that investigated the association between physical activity and pancreatic cancer incidence. Our meta-analysis showed a summary estimate of 0.75 (95% CI 0.64–0.88) for case–control studies (I^2^ = 23%, *n* = 7) and a summary estimate of 0.91 (95% CI 0.86–0.97) for prospective cohort studies (I^2^ = 5%, *n* = 18). Among the six prospective cohort studies that assessed pancreatic cancer mortality, the summary estimate was 1.03 (95% CI 0.83–1.27), I^2^ = 50%. **Conclusions**: Higher levels of physical activity were associated with reduced pancreatic cancer risk. Evidence from a limited number of studies suggests that pre-diagnosis physical activity does not affect pancreatic cancer mortality.

## Simple Summary

Pancreatic cancer is a particularly aggressive form of cancer characterized by poor patient survival, highlighting the need for preventive strategies and interventions to improve long-term survival. While physical activity is known to lower the risk of many different forms of cancer and reduce cancer-specific mortality, its effect on pancreatic cancer is less clear. Therefore, this systematic review and meta-analysis aimed to summarize epidemiological evidence on the relationship between physical activity and pancreatic cancer risk and mortality. A meta-analysis of seven case–control and eighteen prospective cohort studies demonstrated that higher levels of physical activity were associated with a lower risk of developing pancreatic cancer. No significant association was found between pre-diagnosis physical activity and pancreatic cancer mortality, based on data from only six prospective cohort studies. These results could help guide further research and contribute to public health recommendations on physical activity for cancer prevention.

## 1. Introduction

Pancreatic cancer accounted for approximately 3% of all newly diagnosed cancers worldwide in 2022 and was the 6th most common cause of cancer mortality [1]. Despite ongoing developments, pancreatic cancer remains one of the most lethal cancers, with a 5-year overall survival of 13% for all stages combined [2]. The high mortality rate of pancreatic cancer is largely due to early metastasis and late presentation, as the majority of patients have advanced, unresectable disease at initial diagnosis [3]. Therefore, prioritizing strategies aimed at prevention and early detection is crucial.

Modifiable lifestyle risk factors such as smoking, obesity, heavy alcohol consumption, and type-II diabetes have been shown to play an important role in pancreatic cancer etiology [4]. Additionally, physical (in)activity has been hypothesized to influence the risk of developing pancreatic cancer [5,6]. Regular physical activity may decrease the risk of pancreatic cancer through various mechanisms, including beneficial effects on adiposity, insulin sensitivity, chronic low-grade inflammation, immune function, and oxidative stress [6,7]. Specifically, improved insulin sensitivity related to physical activity may be important, as accumulating evidence suggests that impaired glucose tolerance and insulin resistance play a role in pancreatic carcinogenesis [8,9,10]. Physical activity is an effective strategy to reduce insulin resistance, thereby possibly lowering the risk of developing pancreatic cancer. Additionally, physical activity is recognized for its anti-inflammatory effects and is associated with decreased systemic levels of pro-inflammatory biomarkers, such as TNF-α and IL-6, partly due to a reduction in visceral fat mass [6,7]. Chronic low-grade inflammation is emerging as a key mediator of pancreatic cancer development [11].

The preventive effect of physical activity on various cancer types, including colon, breast, endometrial, and gastric cancers, is well-studied and supported by the literature, with relative risk reductions ranging from approximately 10% to 20% [5,6]. Less is known about its role in the risk of pancreatic cancer. Previous meta-analyses have shown a modest association between higher levels of physical activity and a lower incidence of pancreatic cancer [12,13,14,15,16]. However, the evidence grade remains low, and a limited number of studies have examined this relationship [5].

In addition to its influence on cancer risk, physical activity has been linked to reduced cancer-specific mortality in several cancers [17,18]. For pancreatic cancer, the relationship between physical activity and mortality remains unclear, as previous studies have largely focused on pancreatic cancer incidence [12,13,14,15,16].

Therefore, the present systematic review and meta-analysis aimed to summarize epidemiologic evidence assessing the relationship between physical activity and pancreatic cancer risk and mortality.

## 2. Materials and Methods

This systematic review was performed according to the Preferred Reporting Items for Systematic Reviews and Meta-Analyses (PRISMA) 2020 guidelines [19]. The review protocol was registered in the PROSPERO database (registration number: CRD42024594581).

### 2.1. Literature Search

Two authors (M.D.B. and J.E.O.) independently conducted a literature search to identify studies describing the association between physical activity and pancreatic cancer incidence and/or mortality. The electronic databases PubMed and Embase were searched from their earliest available records up to 20 May 2024, using the following keywords: “Pancreas”, “Cancer”, and “Physical activity” and their synonymous terms (Table S1). References of relevant articles were reviewed to supplement the search.

### 2.2. Inclusion and Exclusion Criteria

Articles were eligible for inclusion if the following criteria were met: (1) case–control or prospective cohort studies examining the association between physical activity and pancreatic cancer incidence and/or mortality, (2) outcomes reported adjusted risk estimates (hazard ratio, relative risk, or odds ratio), (3) pancreatic cancer diagnoses or deaths were confirmed through record linkage or medical records, (4) articles were published in English, and (5) full text was available. Conference abstracts and retrospective cohort studies were excluded from our meta-analysis. Retrospective cohort studies were excluded due to the anticipated low number and potential bias from missing data on confounding factors, exposures, or outcomes. When multiple studies reported on the same study population, only the study with the largest sample size was included.

### 2.3. Study Selection

After identifying relevant titles, the abstracts were read, and articles that met the eligibility criteria outlined above were retrieved. Disagreements between the two authors (M.D.B. and J.E.O.) were resolved through discussions between them, with a third author (M.N.) consulted for additional input as needed.

### 2.4. Quality Assessment

The methodological quality and risk of bias of eligible studies were independently assessed by two authors (M.D.B. and J.E.O.) using the Newcastle-Ottawa Scale (NOS). The NOS consists of nine items, categorized into three groups: patient selection, comparability of the study groups, and assessment of exposure and outcome [20]. A score of zero to nine stars was allocated to each study. Studies were classified as low quality (zero to four stars), moderate quality (five to six stars), or high quality (seven to nine stars) according to the NOS score. Any discrepancies were addressed and resolved through discussion.

### 2.5. Data Collection

Data extraction was performed by two authors (M.D.B. and J.E.O.). From each included study, risk estimates (HRs, RRs, and ORs) and their 95% confidence intervals for the highest vs. lowest levels of physical activity in relation to pancreatic cancer incidence and/or mortality were extracted. Additional collected information included the first author’s name, year of publication, study location, study design, number of subjects and cases, duration of follow-up, adjusted factors, and definitions of lowest and highest levels of physical activity. For case–control studies, it was specified whether participants were asked about their recent activity levels (prior to the interview or cancer diagnosis) or about their physical activity during their usual adult life. Physical activity is typically divided into four domains: leisure-time, occupational, transportation, and household. Our analysis focused on leisure-time physical activity, as the predominant focus of epidemiological research lies within this domain. Risk estimates for total physical activity (across more than one physical activity domain) were extracted in case no separate risk estimate for leisure-time physical activity was given. If multiple risk estimates were presented, the most fully adjusted model was selected, and preference was given to moderate-to-vigorous physical activity expressed in metabolic equivalent of task (MET)-hours per week. In studies presenting multiple risk estimates for different time periods, baseline or cumulative (consistent physical activity over time) measurements were selected to reduce heterogeneity.

### 2.6. Statistical Analysis

For the meta-analysis, risk estimates for pancreatic cancer incidence and mortality comparing the highest and lowest levels of physical activity were pooled. When studies provided results stratified for sex without combined results, risk estimates for both males and females were included in the meta-analysis. Since risk ratios do not adhere to a normal distribution, the natural logarithms of the risk estimates [log(risk estimate)] and their corresponding standard errors [SE = [log(upper 95% CI bound of risk estimate) − log(risk estimate)]/1.96] were calculated. A random-effects model was utilized to compute the weighted average of the log(risk estimates), with the inverse-variance method applied for weighing.

Given the higher risk of recall and selection bias in case–control studies [21], we performed separate meta-analyses for case–control and prospective cohort studies. To explore potential sources of heterogeneity, subgroup analyses were conducted when feasible based on the following factors: sex (male, female), geographic region (Asia, Europe, North America), time period for physical activity (recent, usual adult life), and study quality (high, moderate). Moreover, a meta-regression analysis was performed to assess whether studies that adjusted for diabetes mellitus, BMI, and smoking had any independent effect on the summary estimate. In prospective cohort studies, HRs and RRs were pooled without conversion. Due to limited data, accurate conversion of HRs into RRs was not feasible. However, these measures often provide comparable estimates in the context of rare events, such as the incidence or mortality of pancreatic cancer. A stratified analysis was performed to ensure that the summary estimates did not vary by the type of risk estimate.

Heterogeneity among the studies was assessed using the I^2^ statistic, classified as low (<25%), moderate (25–75%), or high (>75%) [22]. Publication bias was evaluated visually by inspecting funnel plots and statistically through Egger’s regression test [23]. Data analysis was conducted using the R-package ‘metafor’ (version 4.6-0) [24]. Two-sided *p* values of less than 0.05 were considered statistically significant.

## 3. Results

### 3.1. Included Studies

**Figure 1:**
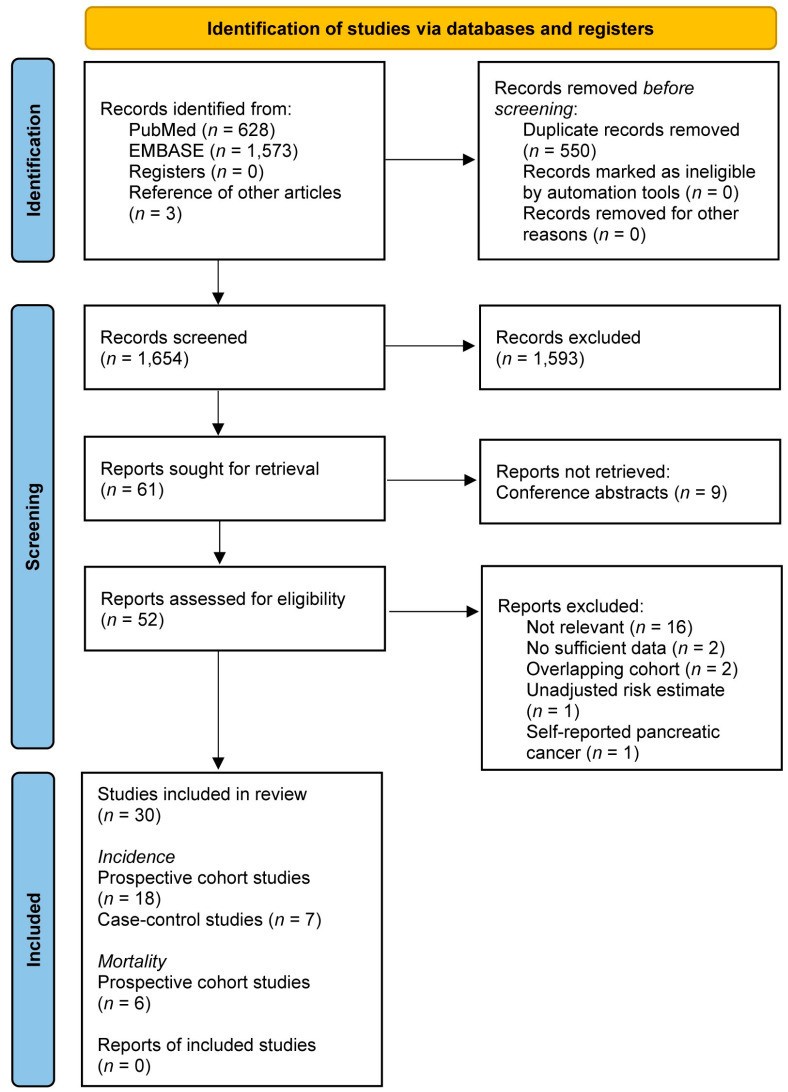
PRISMA flow diagram.

The flowchart of the literature search conducted according to the PRISMA guidelines is presented in Figure 1. A total of 1654 publications were screened by title and/or abstract, of which three were identified by searching the references of relevant studies. Fifty-two of these articles were qualified for full-text evaluation, from which two studies were excluded because the study population represented a sub-cohort of a larger study already included in the present meta-analysis [25,26]. Additionally, one study was excluded because adjusted risk estimates could not be derived [27] and one study was excluded because pancreatic cancer cases were ascertained through self-report [28]. From the study by Michaud et al. [29], only results from the Nurses´ Healthy Study (NHS) cohort were included, as the Healthy Professionals Follow-up Study (HPFS) cohort was already described in the study by Keum et al. [30]. Finally, twenty-five articles on pancreatic cancer incidence and six articles on pancreatic cancer mortality were considered to fit the inclusion criteria for this systematic review and meta-analysis. All authors agreed on including these articles.

### 3.2. Pancreatic Cancer Incidence

#### 3.2.1. Study Characteristics

Eighteen prospective cohort studies [29,30,31,32,33,34,35,36,37,38,39,40,41,42,43,44,45,46] and seven case–control studies [47,48,49,50,51,52,53], describing a total of 3,223,725 participants and 9996 pancreatic cancer incident cases, were included for the pooling of data regarding the association between physical activity and the incidence of pancreatic cancer. These articles were published between 2000 and 2023 and included participants from various regions: North America (*n* = 13) [29,30,32,34,39,40,41,46,48,49,51,52,53], Europe (*n* = 7) [31,33,37,38,42,45,47], and Asia (*n* = 5) [35,36,43,44,50]. Physical activity was measured subjectively by self-report questionnaires or in-person interviews using different types of physical activity questionnaires. Physical activity levels were assessed prior to pancreatic cancer diagnosis in all studies. In prospective cohort studies, physical activity was measured at baseline by asking participants about either their recent or consistent activity levels, with a median follow-up time of 9.8 years (interquartile range 8.0–14.7 years). Case–control studies assessed activity levels either during the subject’s usual adult life [47,48,51,52] or more recently, within 1–2 years before the interview or pancreatic cancer diagnosis [49,50,53]. The majority of studies reported on leisure-time physical activity, whilst seven studies [32,34,38,45,46,52,53] combined activities (usually leisure-time and occupational physical activity) to estimate total physical activity. The cutoff points for the highest and lowest physical activity levels varied considerably across studies. The study characteristics and outcomes are summarized in Table S2. All included studies were of moderate-to-high quality based on the NOS (Table S3). No signs of potential publication bias were observed based on the funnel plot (Figure S1) and Egger´s regression test (*p* = 0.42).

#### 3.2.2. Primary Results

**Figure 2:**
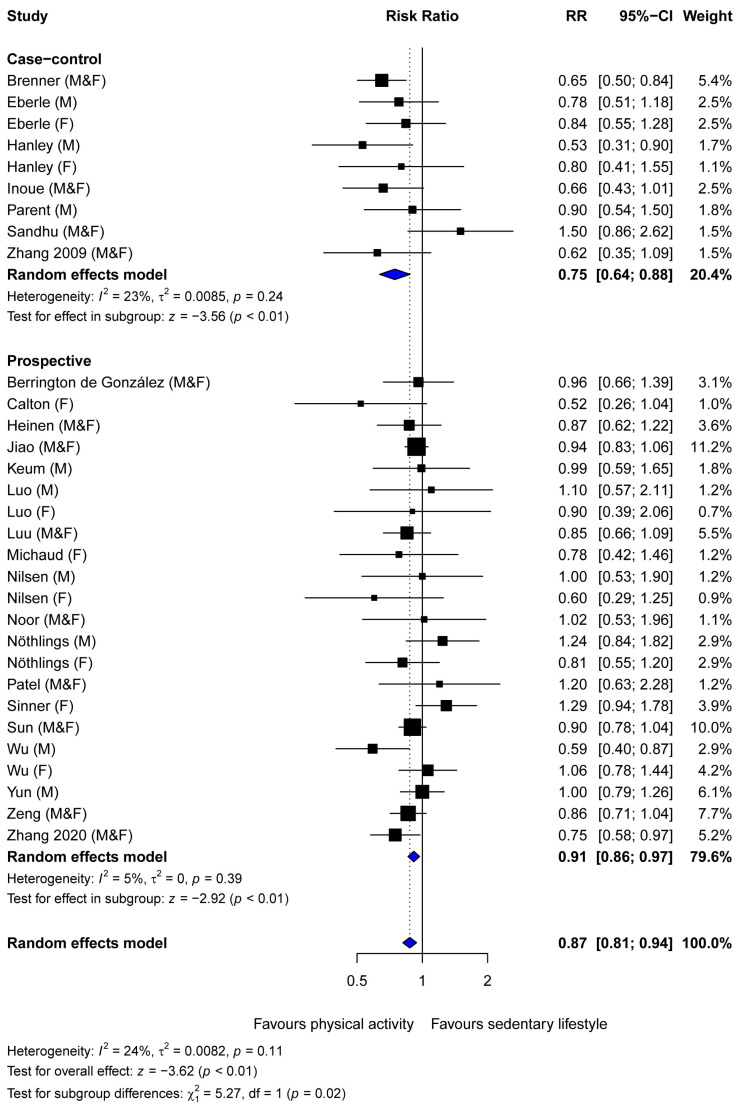
Forest plot of studies examining the association between physical activity and pancreatic cancer incidence by study design [29,30,31,32,33,34,35,36,37,38,39,40,41,42,43,44,45,46,47,48,49,50,51,52,53]. Abbreviations: F, female; M, male.

Our meta-analysis of eighteen prospective cohort studies demonstrated a significant risk reduction in pancreatic cancer among individuals with the highest compared to the lowest levels of physical activity (summary estimate 0.91, 95% CI 0.86–0.97). A stronger decrease in relative risk was observed among seven case–control studies (summary estimate 0.75, 95% CI 0.64–0.88), which was significantly different from the summary estimate observed in prospective cohort studies (P-difference = 0.02, Figure 2). Heterogeneity among the prospective cohort studies (I^2^ = 5% *p* = 0.39) and case–control studies (I^2^ = 23%, *p* = 0.24) was low. The summary estimates did not differ by the type of risk estimate in prospective cohort studies (Table S4). Therefore, RRs and HRs were combined in the analysis. All case–control studies reported ORs.

#### 3.2.3. Subgroup Analysis Results

Subgroup analyses by sex, geographic region, time period for physical activity, and study quality are presented in Table S4. None of these factors showed a significant modifying effect in either prospective cohort or case–control studies. Additionally, in the prospective studies that adjusted for BMI, diabetes mellitus, and/or smoking, these adjustments did not significantly impact the summary estimates in our meta-regression model, with *p* values of 0.86, 0.27, and 0.34, respectively.

### 3.3. Pancreatic Cancer Mortality

#### 3.3.1. Study Characteristics

Data regarding the association between physical activity and mortality from pancreatic cancer were derived from six prospective cohort studies [18,46,54,55,56,57], describing a total of 573,570 participants and 1285 pancreatic cancer deaths. The studies, published between 2003 and 2020, were conducted in various regions: North America (*n* = 3) [18,46,55], Europe (*n* = 1) [54], and Asia (*n* = 2) [56,57]. In all studies, participants subjectively reported their physical activity levels using various self-administered questionnaires. Studies on pancreatic cancer mortality involved cohorts without a pancreatic cancer diagnosis at baseline. Thus, physical activity was assessed at baseline, after which participants were followed from study entry until pancreatic cancer death or the end of the follow-up period. The median follow-up time was 12.1 years (interquartile range 7.5–13.1 years). All studies reported on leisure-time physical activity. The study characteristics and outcomes are shown in Table S5. All included studies were of high quality based on the NOS (Table S3). The funnel plot (Figure S2) and Egger’s regression test (*p* = 0.92) indicated no evidence of potential publication bias.

#### 3.3.2. Primary Results

**Figure 3:**
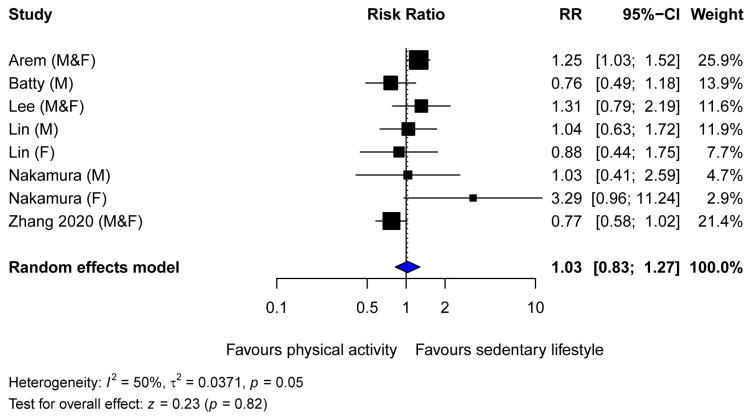
Forest plot of studies examining the association between physical activity and pancreatic cancer mortality [18,46,54,55,56,57]. Abbreviations: F, female; M, male.

No association between higher levels of pre-diagnosis physical activity and mortality from pancreatic cancer was found (summary estimate 1.03, 95% CI 0.83–1.27, Figure 3). There was evidence of moderate heterogeneity (I^2^ = 50%, *p* = 0.05) among the six included prospective cohort studies, largely explained by the study by Nakamura et al., which reported a small number of pancreatic cancer deaths (33 in males and 19 in females) [57]. Subgroup analysis was not performed for the association between physical activity and pancreatic cancer mortality as the number of studies identified was deemed too small (*n* = 6).

## 4. Discussion

The present systematic review and meta-analysis summarizes the latest evidence on the association between physical activity and pancreatic cancer risk and mortality. Our results demonstrate that higher levels of self-reported physical activity are associated with a significant risk reduction in the development of pancreatic cancer (summary estimate 0.75, 95% CI 0.64–0.88 for case–control studies and summary estimate 0.91, 95% CI 0.86–0.97 for prospective cohort studies). When comparing the highest and the lowest categories of physical activity, no association was found between pre-diagnosis physical activity and mortality from pancreatic cancer (summary estimate 1.03, 95% CI 0.83–1.27).

Our literature search yielded six additional studies that described physical activity in relation to the risk of pancreatic cancer that were not included in previous meta-analyses [36,38,42,45,46,52]. The most recent meta-analysis by Xie et al. included only fourteen studies that were published up to 2018 and did not separately analyze case–control and prospective cohort studies [16]. However, case–control studies are generally considered to have a higher risk of recall and selection bias [21], which may explain the stronger inverse association observed between physical activity and reduced pancreatic cancer risk in these studies. Our meta-analysis provides a comprehensive overview of all relevant studies and clearly demonstrates a significant protective effect of physical activity on pancreatic cancer risk in both case–control and prospective cohort studies.

Several biological mechanisms may explain the protective role of physical activity in the development of pancreatic cancer. Physical activity helps maintain a healthy body weight, thereby reducing obesity-related factors that increase the risk of pancreatic cancer, including chronic inflammation and insulin resistance [6,7]. Insulin resistance results in increased concentrations of circulating insulin, which is hypothesized to have tumor-promoting effects in the pancreas, although the exact underlying mechanisms remain unknown [8,58]. Furthermore, acute bouts of exercise have been shown to promote the recirculation of immunoglobulins, anti-inflammatory cytokines, neutrophils, natural killer cells, cytotoxic T-cells, and immature B-cells. Over time, these short-term effects on the immune system enhance immune surveillance and reduce inflammation. However, prolonged and intense exercise can transiently suppress immune function [59]. Another hypothesized mechanism is that physical activity may influence the balance between reactive oxygen species (ROS) and antioxidant defenses, initially promoting oxidative stress but, with repeated physical activity, leading to improved antioxidant defenses [6,60]. Importantly, the association between physical activity and risk of pancreatic cancer appears to be independent of body mass index (BMI) [15,32,33,39,42]. Thus, physical activity may influence the risk of pancreatic cancer by direct exercise-induced effects or indirectly by modulating obesity and type-II diabetes, both of which are closely related to pancreatic cancer risk.

Given the high burden of pancreatic cancer, the reduction in risk associated with regular physical activity could yield substantial public health benefits. However, the optimal type, intensity, duration, and frequency of physical activity necessary for reducing the risk of pancreatic cancer remain largely unclear. Dose–response associations were not analyzed in this meta-analysis due to inconsistent methods for the classification of physical activity levels. Instead, we compared only the highest and lowest physical activity categories. Thus, we recommend that public health initiatives encourage all individuals to meet the recommended levels of physical activity—at least 150 min of moderate- to vigorous-intensity aerobic exercise per week—to improve overall health and reduce the risk of cancer, including pancreatic cancer.

The absence of an association between physical activity and pancreatic cancer mortality in this study could be explained by the fact that pancreatic cancer is a highly fatal disease characterized by late presentation and aggressive tumor biology [3]. Therefore, the beneficial effects of physical activity on the tumor microenvironment, including alterations in tumor metabolism, vascularization, and immune cell infiltration, may not be as evident in pancreatic cancer patients [61,62,63]. Nevertheless, physical activity may influence the stage at which pancreatic cancer is diagnosed. It is currently unknown whether physically active individuals are more likely to present with early stage compared to advanced stage disease in pancreatic cancer. Moreover, limited data exist regarding the influence of physical activity on survival after pancreatic cancer diagnosis [5]. Exercise programs during cancer treatment have been shown to be safe and feasible for patients with pancreatic cancer [64]. Yet, two randomized controlled trials (RCTs) involving patients with advanced-stage pancreatic cancer found no evidence that exercise during chemotherapy impacts oncological survival, although these trials were not specifically powered to evaluate this outcome [65,66]. Ongoing RCTs primarily focus on assessing the feasibility of exercise programs during cancer treatment and their potential to improve physical function and quality of life. It also remains unclear whether different types or intensities of physical activity may have varying effects on pancreatic cancer mortality or survival. Most clinical studies concentrate on aerobic exercise, while resistance training has been shown to be beneficial in managing cancer cachexia, a condition particularly prevalent in pancreatic cancer patients and associated with higher mortality [67,68].

Our meta-analysis has several limitations. First, only studies published in English were considered eligible for inclusion, which may limit the generalizability of our findings. Additionally, focusing solely on published studies raises concerns about publication bias. Given the rarity of pancreatic cancer, smaller studies are common, and these may be less likely to report negative or inconclusive findings, potentially skewing the conclusions of our meta-analysis. Nevertheless, both the funnel plot and Egger’s regression test showed no signs of potential publication bias. Another limiting factor is the fact that all included studies collected physical activity data through self-report questionnaires or in-person interviews, introducing the possibility of measurement errors due to recall bias. None of the studies included in our meta-analysis have used wearable activity trackers to measure physical activity, while this approach is likely to provide higher reliability and validity compared to self-report methods [69]. Moreover, definitions of the highest and lowest physical activity levels were inconsistent across studies, thus reducing comparability between studies. Risk estimates expressed in units of MET hours per week were selected as much as possible, but physical activity assessment methods remained heterogeneous. Furthermore, established risk factors, such as smoking, obesity, type II diabetes, and a family history of pancreatic cancer, were not adjusted for in all studies. An additional limitation is that none of the studies examining pancreatic cancer mortality accounted for the stage of the disease or the treatment received, both of which could significantly impact mortality rates. Finally, only a small number of studies provided information on physical activity and mortality from pancreatic cancer. Therefore, the evidence is still too limited to draw precise conclusions about physical activity in relation to pancreatic cancer mortality.

Future studies should standardize results by reporting self-reported physical activity in MET hours per week to reduce methodological heterogeneity between studies and allow an estimation of dose–response associations. Ideally, wearable activity trackers should be used to measure physical activity in a more objective manner. Moreover, there is a need for additional studies assessing physical activity in relation to the stage of pancreatic cancer at diagnosis, mortality from pancreatic cancer, and survival after pancreatic cancer diagnosis.

## 5. Conclusions

In conclusion, the results of the present systematic review and meta-analysis indicate that higher levels of physical activity are associated with a reduced risk of developing pancreatic cancer. No relationship was demonstrated between physical activity and mortality from pancreatic cancer, but evidence remains limited mainly due to insufficient research in this area. Recognizing that physical activity is a modifiable behavior, our findings provide an opportunity to reduce the population’s risk of pancreatic cancer and underscore the importance of promoting physical activity for overall health and cancer prevention.

## Data Availability

The data presented in this study are publicly available by applying the search strategy outlined in this manuscript to PubMed and Embase.

## Supplementary Materials

The following supporting information can be downloaded at: https://www.mdpi.com/article/10.3390/cancers16213594/s1, Figure S1: Funnel plot of studies on pancreatic cancer incidence; Figure S2: Funnel plot of studies on pancreatic cancer mortality; Table S1: Search strategy; Table S2: Included studies describing the relationship between physical activity and pancreatic cancer incidence; Table S3: Quality assessment by the Newcastle-Ottawa Scale. Table S4: Subgroup meta-analyses of the association between physical activity and pancreatic cancer incidence; Table S5: Included studies describing the relationship between physical activity and pancreatic cancer mortality.
