# Supplementary material for: Association Between Physical Activity and Pancreatic Cancer Risk and Mortality: A Systematic Review and Meta-Analysis
Source: Cancers (Basel). 2024 Oct 24;16(21):3594. doi: 10.3390/cancers16213594 (PMC11544951; doi:10.3390/cancers16213594)
Supplement: Supplementary file 1 [file cancers-16-03594-s001.zip › cancers-3250434-supplementary.pdf]

Table S1. Search strategy.

PubMed and Embase were searched from their earliest available records up to May 20, 2024.

**Database**	**Search strategy**
PubMed	("Pancreas"[Mesh] OR pancreas[tiab] OR pancreatic[tiab])<br>AND<br>("Pancreatic Neoplasms"[Mesh] OR "Neoplasms"[Mesh] OR cancer*[tiab] OR carcinoma*[tiab] OR adenocarcinoma*[tiab] OR tumour*[tiab] OR tumor*[tiab] OR neoplasm*[tiab])<br>AND<br>("Exercise"[Mesh] OR physical activity[tiab] OR exercise*[tiab] OR aerobic activity*[tiab] OR physical status[tiab] OR physical training*[tiab])
EMBASE	('pancreas'/exp OR pancreas:ti,ab,kw OR pancreatic:ti,ab,kw)<br>AND<br>('pancreas cancer'/exp OR 'malignant neoplasm'/exp OR cancer*:ti,ab,kw OR carcinoma:ti,ab,kw OR adenocarcinoma*:ti,ab,kw OR tumour*:ti,ab,kw OR tumor*:ti,ab,kw OR neoplasm*:ti,ab,kw)<br>AND<br>('exercise'/exp OR 'physical activity'/exp OR 'physical activity*':ti,ab,kw OR exercise*:ti,ab,kw OR 'aerobic activity*':ti,ab,kw OR 'physical status*':ti,ab,kw OR 'physical training*':ti,ab,kw)

Table S2. Included studies describing the relationship between physical activity and pancreatic cancer incidence.

**Author, year**	**Sex**	**Study location**	**Subjects/ controls**	**Cases**	**Follow-up (years)**	**PA domain**	**Timing of PA** [a]	**Low PA defined by**	**High PA defined by**	**Risk estimate (95% CI) High vs. low**	**Adjustment factors**
Prospective cohort studies											
Berrington de González, 2006 [31]	M&F	Europe	438,405	324	6.7	Leisure-time	NA	First quartile of METs	Fourth quartile of METs	0.96 (0.66-1.39)	Age, sex, country, smoking, diabetes
Calton, 2008 [32]	F	USA	33,530	70	8.5	Total	NA	34.0–50.1 MET hours/day	63.44–100.2 MET hours/day	0.52 (0.26-1.05)	Age, BMI, race, smoking status, history of diabetes, height
Heinen, 2011 [33]	M&F	The Netherlands	4,438	408	13.3	Leisure-time	NA	<30 min/day	≥90 min/day	0.87 (0.62-1.22)	Age, sex, smoking, BMI, energy intake, intake of vegetables
Jiao, 2009 [34]	M&F<br>M<br>F	USA	450,416<br>263,398<br>187,018	1,057<br>675<br>382	7.2	Total	NA	<3-4 times per week	≥3-4 times per week	0.94 (0.83-1.06)<br>0.93 (0.80-1.08)<br>0.94 (0.77-1.16)	Age, sex, race, educational level, marital status, total energy intake, smoking, alcohol use, dietary quality, BMI
Keum, 2016 [30]	M	USA	43,479	235	15.8	Leisure-time	NA	≤8.9 MET hours/week	≥63 MET hours/ week	0.99 (0.59-1.64)	Age, questionnaire cycle, race, smoking, family history of cancer, history of physical examination for screening purpose, current aspirin use, current multivitamin use, intakes of total calories, alcohol, red and processed meat, whole grains, fruits and vegetables, BMI, history of diabetes

Luo, 2007 [35]	M<br>F	Japan	47,499<br>52,171	128<br>96	11	Leisure-time	NA	<1 day/week	>2 days/week	1.1 (0.6–2.2)<br>0.9 (0.4–2.1)	Age, study area, smoking status, history of diabetes, BMI, alcohol intake, history of cholelithiasis
Luu, 2022 [36]	M&F	Singapore	61,321	316	17.7	Leisure-time	NA	No weekly PA	Weekly PA	0.85 (0.66–1.09)	Age, sex, dialect, year of enrollment, education level, diabetes history, total energy intake, smoking status, BMI, Alternative Healthy Eating Index-2010, sleep
Michaud, 2001 [29]	F	USA	77,559	110	9.7	Leisure-time	NA	First quintile: ≤2 MET hours/week	Fifth quintile: ≥21.8 MET hours/week	0.78 (0.42-1.47)	Age, smoking status, history of diabetes, history of cholecystectomy
Nilsen, 2000 [37]	M<br>F	Norway	31,000<br>32,374	96<br>70	9.8	Leisure-time	NA	<1 time/week	>3 times per week	1.0 (0.5-1.8)<br>0.6 (0.3-1.3)	Age
Noor, 2016 [38]	M&F	UK	4,058	88	8.6	Total	NA	Sedentary job and no leisure-time activity	Sedentary job with >1.0 hours leisure-time activity per day or standing job with >0.5 hours leisure-time activity per day or physical job with at least some leisure-time activity or heavy manual job	1.02 (0.53-1.96)	Age, sex, smoking, diabetes, BMI

Nöthlings, 2007 [39]	M<br>F	USA	77,255<br>90,175	237<br>235	8	Leisure-time	NA	First quartile of METs	Fourth quartile of METs	1.24 (0.85-1.84)<br>0.81 (0.55-1.20)	Smoking status, pack-years of smoking, family history of pancreatic cancer, history of diabetes, age at cohort entry, energy intake, intake of red meat, processed meat, BMI
Patel, 2005 [40]	M&F<br>M<br>F	USA	145,627<br>69,589<br>76,038	242<br>137<br>105	7	Leisure-time	NA	0 MET hours per week	>31.5 MET hours per week	1.20 (0.63-2.27)<br>1.01 (0.39-2.60)<br>1.42 (0.59-3.41)	Age, sex, smoking status, years since quitting smoking among former smokers, education, family history of pancreatic cancer, personal history of gallbladder disease, personal history of diabetes, height, total caloric intake, MET hours per week in 1992, BMI
Sinner, 2005 [41]	F	USA	38,002	209	14.7	Leisure-time	NA	Low PA level	High PA level	1.29 (0.93-1.77)	Age, smoking status, multivitamin use
Sun, 2022 [42]	M&F<br>M<br>F	Norway, Sweden	570,021<br>285,093<br>284,928	1,389<br>764<br>625	20	Leisure-time	NA	Low PA: sedentary to light PA	High PA: moderate to hard PA	0.90 (0.78-1.04)<br>0.89 (0.74-1.06)<br>0.90 (0.70-1.17)	Sex, cohort, baseline age, date of birth, smoking status and intensity, BMI
Wu, 2018 [43]	M<br>F	China	60,037<br>72,451	159<br>225	10.3<br>16.1	Leisure-time	NA	No PA	>1.07 MET hours/day/year	0.59 (0.40-0.87)<br>1.06 (0.78-1.44)	Age, education, income, waist-to-hip ratio, smoking status, alcohol consumption, family history of pancreatic cancer, gallstone history, gallbladder surgery history, diabetes history

Yun, 2008 [44]	M	Korea	444,936	349	7.3	Leisure-time		Low PA (≤ 4 times/week for <30 min/session or ≤1 time/week for ≥30 min/session)	Moderate-High PA, defined as:<br>- Moderate: 2–4 times/week for ≥30 min/session or ≥5 times/week for <30 min/session<br>- High: ≥5 times/week for ≥30 min/session	1.00 (0.81-1.24)	Age, dietary preference, smoking status, amount of alcohol drinking, BMI, employment, fasting blood sugar
Zeng, 2023 [45]	M&F<br>M<br>F	UK	340,631	1,129<br>659<br>470	13.1	Total	NA	0 days per week moderate or vigorous activity (10+ minutes)	≥6 days per week moderate activity (10+ minutes) or ≥3 days per week vigorous physical activity (10+ minutes)	0.86 (0.71-1.04)<br>0.84 (0.66-1.08)<br>0.88 (0.66-1.19)	Age, sex, education level, socioeconomic status, first 5 principal components of ancestry
Zhang, 2020 [46]	M&F	USA	95,962	337	8.87	Total	NA	<75 min/week	≥150 min/week	0.75 (0.58-0.98)	Age, sex, race, educational degree, smoking status, aspirin use, history of diabetes, family history of pancreatic cancer, total energy intake
Case-control studies											
Brenner, 2014 [47]	M&F<br>M<br>F	Czech Republic and Slovakia	930<br>500<br>430	826<br>468<br>358	NS	Leisure-time	Past	Sedentary: <26 hours/year	Non-sedentary: ≥26 hours/year	0.65 (0.52-0.87)<br>0.74 (0.54-1.01)<br>0.53 (0.38-0.75)	Age, sex, center, education level, alcohol intake, tobacco smoking, BMI, history of diabetes, hypertension, gallstones, pancreatitis

Eberle, 2005 [48]	M<br>F	USA	883<br>818	291<br>241	NS	Leisure-time	Past	PA <1 time/month	Daily or almost daily PA	0.78 (0.52–1.2)<br>0.84 (0.56–1.3)	Males: age, education and cigarette smoking status<br>Females: age
Hanley, 2001 [49]	M<br><br><br><br>F	Canada	1,505<br><br><br><br>1,414	173<br><br><br><br>139	NS	Leisure-time	Recent	Composite index of moderate and vigorous PA: <7.1118<br>Composite index of moderate and vigorous PA: <6.11	Composite index of moderate and vigorous PA: ≥36.66<br><br>Composite index of moderate and vigorous PA: ≥28.83	0.53 (0.31–0.90)<br><br><br><br>0.80 (0.41–1.54)	Males: age, province, caloric intake, percent change in weight, maximum BMI<br><br>Females: age, province of residence, cigarette pack-years and age at menarche
Inoue, 2003 [50]	M&F<br>M<br>F	Japan	2,000<br>1,220<br>780	200<br>122<br>78	NS	Leisure-time	Recent	<2 times/week	≥2 times/week	0.66 (0.43–1.01)<br>0.73 (0.42–1.27)<br>0.55 (0.27–1.10)	Age, sex, family history of pancreatic cancer, past/present history of diabetes, bowel habits, raw vegetable intake, alcohol drinking
Parent, 2011 [51]	M	Canada	533	116	NS	Leisure-time	Past	Leisure-time PA <once a week during adult life	Leisure-time PA ≥once a week for at least 6 months during adult life	0.90 (0.54-1.50)	Age, socio-economic status, educational level, ethnicity, respondent status, smoking, BMI, coffee consumption, alcohol consumption, b-carotene index, occupational physical activity levels
Sandhu, 2020 [52]	M&F<br>M<br>F	Canada	1,254<br>666<br>588	315<br>162<br>153	NS	Total	Past	Inactive at all ages	Persistent high activity	1.50 (0.86-2.62)<br>1.78 (0.83-3.80)<br>1.24 (0.51-3.04)	Age, sex, education, race, alcohol intake, smoking, fruit, vegetable and meat consumption, family history of pancreatic cancer

Zhang, 2009 [53]	M&F	USA	554	186	NS	Total	Recent	First quartile PA	Fourth quartile PA	0.62 (0.35-1.09)	Age, sex, race, education, cigarette smoking, alcohol intake and intakes of energy, fat, fiber, fruits and vegetables

[a] Recent activity: less than two years before the diagnosis or interview; past activity: during someone's usual adult life.
Abbreviations: BMI, body mass index; CI, confidence interval; F, females; M, males; MET, metabolic equivalent of task; PA, physical activity; NA, not applicable; NS, not specified.

Table S3. Quality assessment by the Newcastle-Ottawa Scale.

Study, year	Selection ****				Comparability [a] **		Outcome/Exposure ***			Total score	Study quality [b]
	1	2	3	4	5	6	7	8	9		
Incidence – Prospective cohort studies											
Berrington de González, 2006 [31]	*	*	0	*	*	*	*	*	*	8	High
Calton, 2008 [32]	*	*	0	*	*	*	*	*	*	8	High
Heinen, 2011 [33]	*	*	0	*	*	*	*	*	*	8	High
Jiao, 2009 [34]	*	*	0	*	*	*	*	*	*	8	High
Keum, 2016 [30]	0	*	0	*	*	*	*	*	*	7	High
Luo, 2007 [35]	*	*	0	*	*	*	*	*	*	8	High
Luu, 2022 [36]	*	*	*	*	*	*	*	*	*	9	High
Michaud, 2001 [29]	0	*	0	*	*	*	*	*	0	6	Moderate
Nilsen, 2000 [37]	*	*	0	*	*	*	*	*	*	8	High
Noor, 2016 [38]	*	*	0	*	*	*	*	*	*	8	High
Nöthlings, 2007 [39]	*	*	0	*	*	*	*	*	*	8	High
Patel, 2005 [40]	*	*	0	*	*	*	*	*	*	8	High
Sinner, 2005 [41]	*	*	0	*	*	*	*	*	*	8	High

Sun, 2022 [42]	*	*	0	*	*	*	*	*	*	8	High
Wu, 2018 [43]	*	*	*	*	*	*	*	*	*	9	High
Yun, 2008 [44]	*	*	0	*	*	*	*	*	*	8	High
Zeng, 2023 [45]	*	*	**0**	*	*	*	*	*	*	8	High
Zhang, 2020 [46]	*	*	0	*	*	*	0	*	0	6	Moderate
Incidence – Case-control studies											
Brenner, 2014 [47]	*	*	*	*	*	*	0	*	*	8	High
Eberle, 2005 [48]	*	*	*	*	*	*	0	*	*	8	High
Hanley, 2001 [49]	*	*	*	*	*	*	0	*	0	7	High
Inoue, 2003 [50]	*	*	0	*	*	*	0	*	*	7	High
Parent, 2011 [51]	*	*	*	*	*	*	0	*	*	8	High
Sandhu, 2020 [52]	*	*	*	*	*	*	0	*	0	7	High
Zhang, 2009 [53]	*	*	*	*	*	*	0	*	*	8	High
Mortality – Prospective cohort studies											
Arem, 2014 [18]	*	*	0	*	*	*	*	*	*	8	High
Batty, 2009 [54]	0	*	0	*	*	*	*	*	*	7	High
Lee, 2003 [55]	0	*	0	*	*	*	*	*	*	7	High
Lin, 2007 [56]	*	*	0	*	*	*	*	*	*	8	High
Nakamura, 2011 [57]	*	*	0	*	*	*	*	*	*	8	High
Zhang, 2020 [46]	*	*	0	*	*	*	*	*	0	7	High

[a] **Comparability was scored by assigning 1 star when the study controlled for age and another star when the study controlled for sex.**

[b] **Study quality was classified as low (zero to four stars), moderate (five to six stars) or high (seven to nine stars).**

Table S4. Subgroup meta-analyses of the association between physical activity and pancreatic cancer incidence [29-53].

	**Prospective studies**				**Case-control studies**			
**Factor**	**No. of estimates**	**Summary estimate (95% CI)**	**I²**	**P value for subgroup difference**	**No. of estimates**	**Summary estimate (95% CI)**	**I²**	**P value for subgroup difference**
**Effect measure**								
Hazard ratio	12	0.89 (0.82-0.97)	18%	0.36	NA			
Relative risk	10	0.94 (0.86-1.03)	0%		NA			
**Sex**								
Female	11	0.94 (0.84-1.05)	5%	0.76	6	0.78 (0.64-0.94)	29%	0.52
Male	10	0.93 (0.87-0.99)	1%		5	0.69 (0.52-0.92)	25%	
**Region of origin**								
Asia	6	0.90 (0.75-1.07)	28%	0.53	1	0.66 (0.43-1.01)	NA	0.39
Europe	7	0.87 (0.78-0.97)	0%		1	0.65 (0.5-0.84)	NA	
North-America	9	0.94 (0.86-1.03)	26%		7	0.81 (0.65-1.01)	27%	
**Time period for physical activity**								
Recent	NA				4	0.64 (0.49-0.83)	48%	
Past	NA				5	0.84 (0.65-1.08)	0%	0.14
**Study quality**								
High	20	0.93 (0.87-0.99)	3%	0.11	9	0.75 (0.64-0.88)	23%	NA
Moderate	2	0.75 (0.59-0.96)	0%		0	NA	NA	NA

Abbreviations: CI, confidence interval; NA, not applicable.

Table S5. Included studies describing the relationship between physical activity and pancreatic cancer mortality.

Author, year	Sex	Study location	Subjects/ controls	Cases	Follow-up (years)	PA domain	Low PA defined by	High PA defined by	Risk estimate (95% CI) High vs. low	Adjustment factors
Prospective cohort studies										
Arem, 2014 [18]	M&F	USA	293,511	149	12.1	Leisure-time	Never/rarely	>7 hours/week	1.25 (1.03–1.53)	Age, sex, BMI, education, race, alcohol, Healthy Eating Index-2010 score, calories, marriage status, diabetes, smoking
Batty, 2009 [54]	M	UK	17,898	163	38 (maximum)	Leisure-time	Inactive	Active	0.76 (0.49-1.18)	BMI, plasma cholesterol, socio-economic status, diabetes/blood glucose, marital status, forced expiratory volume in 1 second, height, age at risk, smoking, diastolic and systolic blood pressure
Lee, 2003 [55]	M&F	USA	32,687	212	8.1	Leisure-time	Expending <2100 kJ/week	Expending ≥10 500 kJ/week	1.31 (0.69–1.92)	Age, sex, cigarette smoking, diabetes.
Lin, 2007 [56]	M<br>F	Japan	43,579<br>59,107	207<br>195	12.8	Leisure-time	<1 hours of sports/week	≥5 hours of sports/week	1.04 (0.63–1.72)<br>0.88 (0.44–1.74)	Age, BMI, cigarette smoking, history of diabetes, history of gallbladder diseases
Nakamura, 2011 [57]	M<br>F	Japan	14,241<br>16,585	33<br>19	6.9	Leisure-time	First tertile of MET hours/week	Third tertile of MET hours/week	1.03 (0.41–2.60)<br>3.29 (0.96–11.2)	Age, smoking status, BMI, history of diabetes

Zhang, 2020 [46]	M&F	USA	95,962	307	13.34	Leisure-time	<75 min/week	≥150 min/week	0.77 (0.58-1.01)	Age, sex, race, educational degree, smoking status, aspirin use, history of diabetes, family history of pancreatic cancer, total energy intake

Abbreviations: BMI, body mass index; CI, confidence interval; F, females; M, males; MET, metabolic equivalent of task; PA, physical activity.

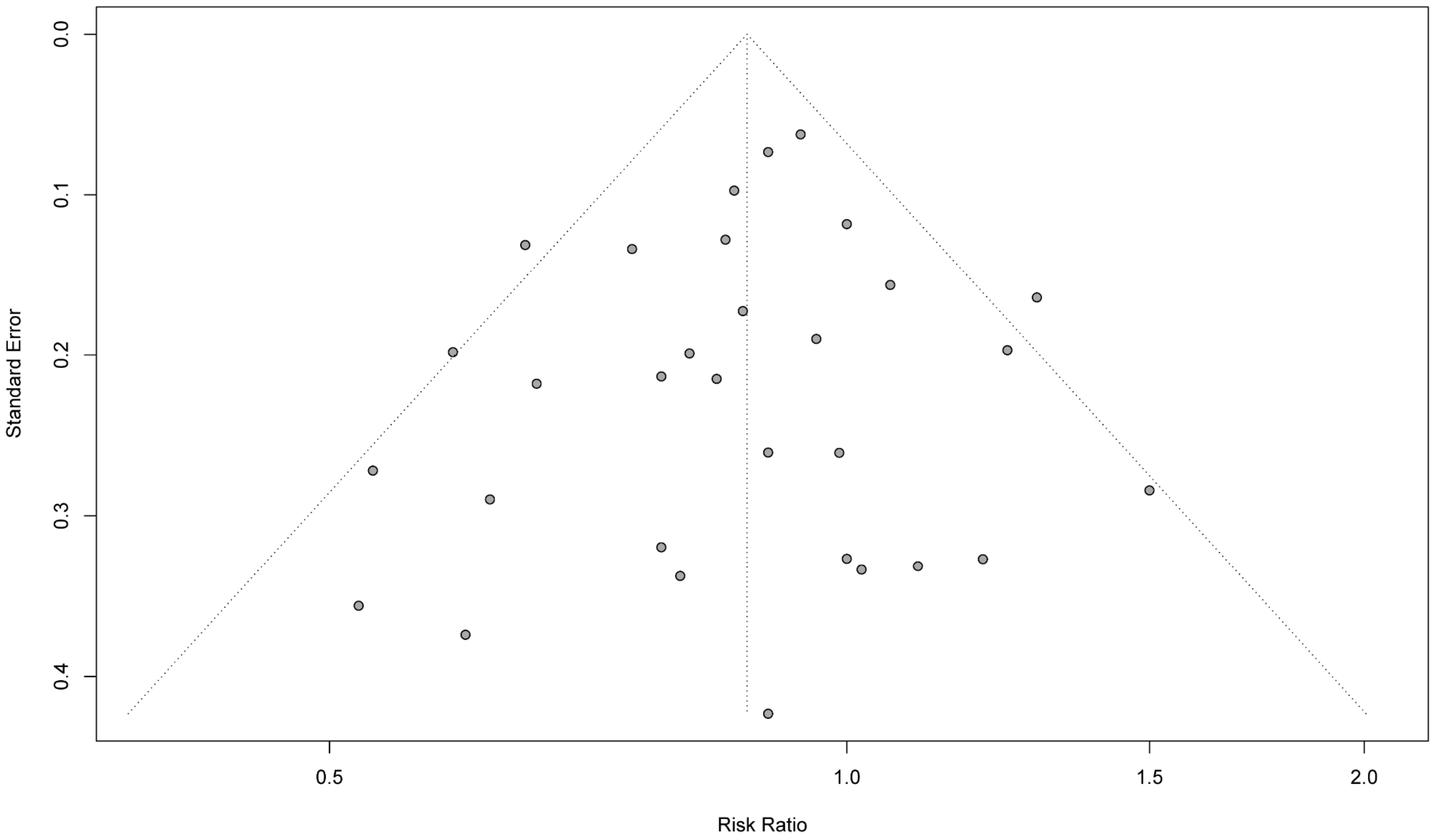

Figure S1: Funnel plot of studies on pancreatic cancer incidence.

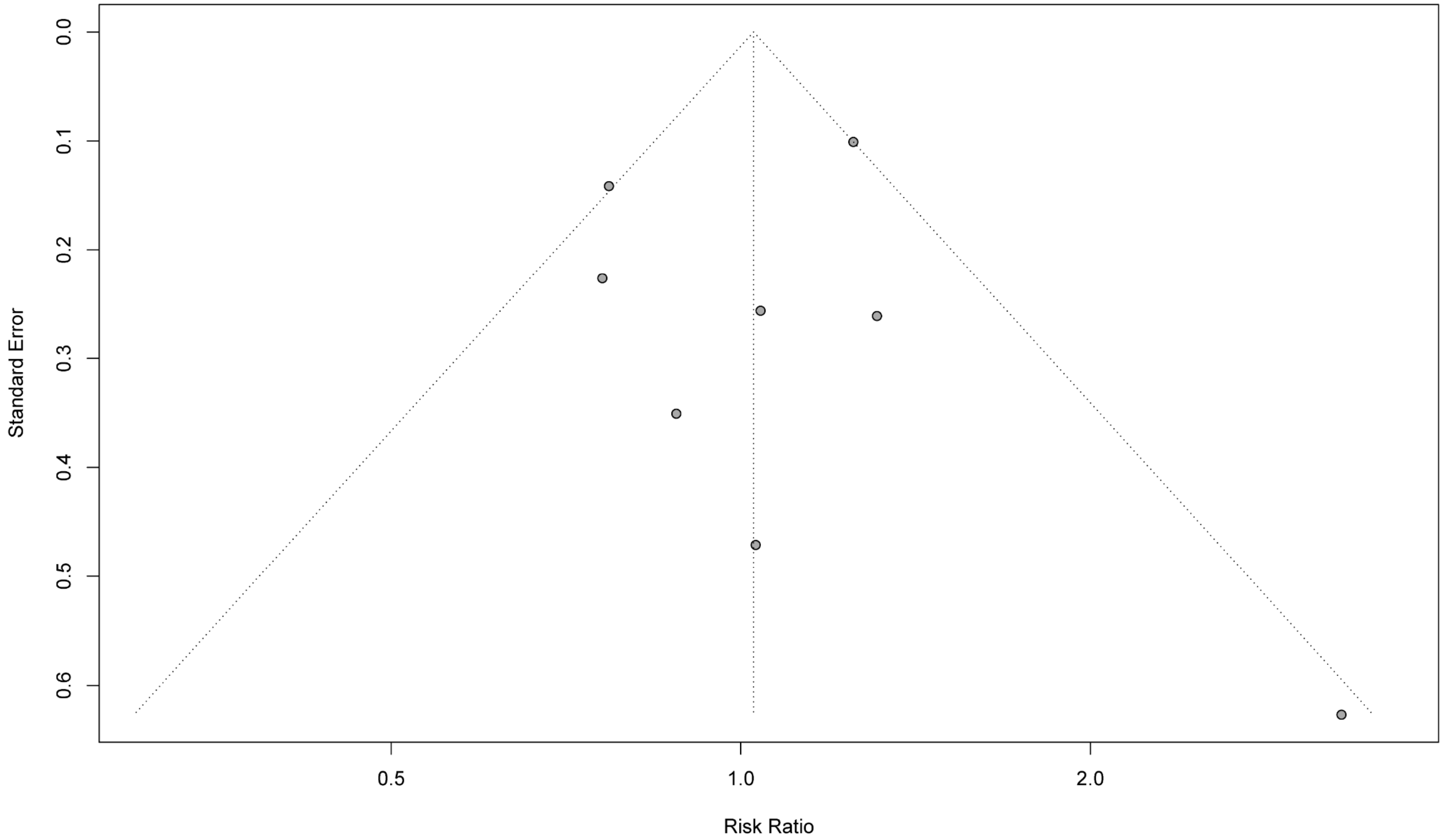

Figure S2: Funnel plot of studies on pancreatic cancer mortality
